# Thioredoxins function as deglutathionylase enzymes in the yeast *Saccharomyces cerevisiae*

**DOI:** 10.1186/1471-2091-11-3

**Published:** 2010-01-14

**Authors:** Darren Greetham, Jill Vickerstaff, Daniel Shenton, Gabriel G Perrone, Ian W Dawes, Chris M Grant

**Affiliations:** 1 The University of Manchester, Faculty of Life Sciences, Manchester M13 9PT, UK; 2 School of Biotechnology and Biomolecular Sciences, University of New South Wales, Sydney, NSW 2052, Australia

## Abstract

**Background:**

Protein-SH groups are amongst the most easily oxidized residues in proteins, but irreversible oxidation can be prevented by protein glutathionylation, in which protein-SH groups form mixed disulphides with glutathione. Glutaredoxins and thioredoxins are key oxidoreductases which have been implicated in regulating glutathionylation/deglutathionylation in diverse organisms. Glutaredoxins have been proposed to be the predominant deglutathionylase enzymes in many plant and mammalian species, whereas, thioredoxins have generally been thought to be relatively inefficient in deglutathionylation.

**Results:**

We show here that the levels of glutathionylated proteins in yeast are regulated in parallel with the growth cycle, and are maximal during stationary phase growth. This increase in glutathionylation is not a response to increased reactive oxygen species generated from the shift to respiratory metabolism, but appears to be a general response to starvation conditions. Our data indicate that glutathionylation levels are constitutively high in all growth phases in thioredoxin mutants and are unaffected in glutaredoxin mutants. We have confirmed that thioredoxins, but not glutaredoxins, catalyse deglutathionylation of model glutathionylated substrates using purified thioredoxin and glutaredoxin proteins. Furthermore, we show that the deglutathionylase activity of thioredoxins is required to reduce the high levels of glutathionylation in stationary phase cells, which occurs as cells exit stationary phase and resume vegetative growth.

**Conclusions:**

There is increasing evidence that the thioredoxin and glutathione redox systems have overlapping functions and these present data indicate that the thioredoxin system plays a key role in regulating the modification of proteins by the glutathione system.

## Background

All aerobic organisms are exposed to reactive oxygen species (ROS) during the course of normal aerobic metabolism or following exposure to radical-generating compounds. Such ROS cause wide-ranging damage to macromolecules, resulting in genetic degeneration and physiological dysfunction, leading eventually to cell death. Cysteine residues are one of the most easily oxidized residues in proteins, and oxidation can result in intermolecular protein cross-linking and enzyme inactivation. However, such irreversible oxidation events can be prevented by protein S-thiolation, in which protein -SH groups form mixed disulphides with low molecular weight thiol compounds [1,2].

**Figure 1:**
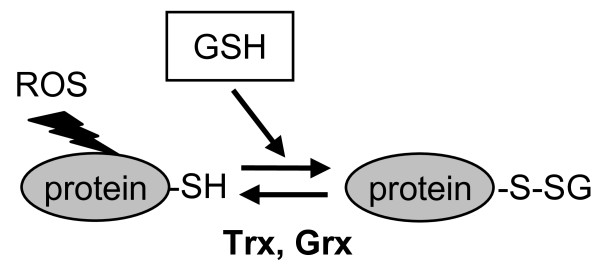
**Modification of proteins by glutathionylation**. This reversible post-translational modification involves the formation of a mixed disulphide between a free thiol group on a protein and a molecule of glutathione. This may occur through oxidation of a protein-thiol group in response to ROS, and reaction with GSH as shown in the diagram. Alternatively, oxidized GSSG may react with protein-SH groups (for a review, see [9]). Deglutathionylation may be catalysed by glutaredoxin (Grx) or thioredoxin (Trx).

Glutathionylation is the major form of S-thiolation in eukaryotic cells. This reversible post-translational modification involves the formation of a mixed disulphide between a free thiol on a protein and a molecule of glutathione (GSH) (Fig. 1). It is particularly important since it can both protect cysteine residues from irreversible oxidation and can also regulate the activity of many target proteins. Greater than 150 targets of modification have been identified from eukaryotic species affecting diverse processes including glycolysis, protein synthesis, protein degradation, signal transduction and transport [3,4]. In many cases, this protein modification is implicated in the regulation of protein function and activity; examples include the HIV-1 protease [5], ubiquitin-conjugating enzymes in bovine retina cells [6], DNA binding by the transcription factor c-Jun [7] and the glycolytic enzyme glyceraldehyde-3-phosphate dehydrogenase (GAPDH) [8]. Protein glutathionylation is a dynamic process that occurs in cells under physiological conditions, as well as following exposure to an oxidative stress. Models have been proposed in which this protein modification does not require an enzymatic activity, but proceeds via the reaction of partially oxidised protein sulphydryls with GSH, or by thiol/disulphide exchange reactions with the oxidised disulphide form of glutathione (Fig. 1) [9]. There does not appear to be any unifying feature of target proteins, and the fact that not all -SH containing proteins are modified in response to an oxidative stress suggests that this protein modification must be tightly regulated [10,11].

To protect protein-SH groups against irreversible oxidation, or to serve a regulatory function, glutathionylation must be reversible. Many studies have demonstrated that modified proteins formed during oxidative stress are readily deglutathionylated once the stress is removed, but the physiological electron donors are unclear. Three main classes of enzyme have been implicated in this reaction, namely sulphiredoxin, glutaredoxins and thioredoxins [9]. Sulphiredoxin is an oxidoreductase which was originally identified based on its ability to reduce cysteine sulphinic acid in 2-Cys peroxiredoxins. The human enzyme has been proposed to act as a deglutathionylating enzyme [12], although the specificity of this reaction has been questioned [9]. Glutaredoxins and thioredoxins were originally identified as hydrogen donors for ribonucleotide reductase, but also act upon a number of metabolic enzymes that form a disulphide as part of their catalytic cycle. They are structurally similar and have been conserved throughout evolution. Despite considerable functional overlap, they are differentially regulated. The oxidised disulphide form of thioredoxin is reduced directly by NADPH and thioredoxin reductase, whereas, glutaredoxin is reduced by glutathione (GSH) using electrons donated by NADPH. Glutaredoxins appear to be the most efficient deglutathionylase enzymes based on *in vitro *experiments. For example, a correlation has been demonstrated between protein-SSG reduction and glutaredoxin activity in mammalian cells [13]. Additionally, mammalian mitochondrial glutaredoxin 2 has been implicated in protein glutathionylation, catalysing the formation of protein mixed disulphides with glutathione [14]. Thioredoxins have also been implicated in deglutathionylation in *in vitro *experiments, but the physiological relevance of this reaction is unclear [4,15].

Yeast, like most eukaryotes, contains a complete cytoplasmic thioredoxin system, comprising two thioredoxins (*TRX1-2*) and a thioredoxin reductase (*TRR1*), which functions in protection against oxidative stress [reviewed in [16]]. Trx1 and Trx2 are active as antioxidants and play key roles in protection against oxidative stress induced by various ROS [17]. Two yeast genes encode classical glutaredoxins (*GRX1 *and *GRX2*). Grx1 and Grx2 are active as GSH-dependent oxidoreductases, but appear to have distinct cellular functions [18,19]. Dithiol glutaredoxins have been proposed to be the predominant deglutathionylase enzymes in many organisms as described above. However, we have previously shown that the global levels of protein S-thiolation are unaffected in yeast mutants lacking glutaredoxins (*GRX1 *and *GRX2) *and are elevated in mutants lacking thioredoxins (*TRX1 *and *TRX2*) [17,18]. In this current study we have examined the roles of glutaredoxins and thioredoxins in the control of glutathionylation. Our data show that thioredoxins, but not glutaredoxins, are required to maintain glutathionylation levels during the yeast growth cycle. Furthermore, we show that thioredoxins, but not glutaredoxins, are active in the deglutathionylation of model mixed disulphide substrates *in vitro*. The deglutathionylase activity of thioredoxins appears to be particularly required in stationary phase cells and we propose that thioredoxins function to reduce glutathionylated-proteins as cells exit stationary phase and resume vegetative growth.

## Results

### Glutathionylation is growth phase regulated in yeast

**Figure 2:**
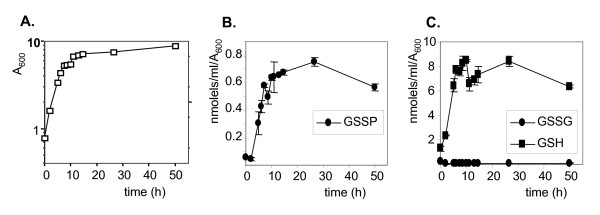
**Glutathionylation is growth phase regulated in yeast**. **(A) **The wild-type strain was grown in minimal SD media and growth monitored by following A_600_. **(B) **Protein bound (GSSP), reduced (GSH) **(C)**, and oxidized (GSSG) **(C) **glutathione levels were quantified at intervals as indicated. Values shown are the means of at least three independent determinations and are given in nmol/ml/A_600_.

Yeast cells grow by fermentation on glucose-based media, until the glucose is exhausted; they then shift to growth by respiration using ethanol generated from glycolysis. The diauxic shift occurs when cells exhaust the glucose and there is a transient growth lag as they switch from fermentation to growth by respiration. Cells then arrest growth and enter stationary phase when there is no longer any net increase in cell number. Yeast cell growth was followed on minimal media (Fig. 2A) and glutathione levels measured. Glutathionylated-protein (GSSP) levels were found to be lowest during exponential phase growth, and increased as cells progressed through the diauxic shift and entered stationary phase (Fig. 2B). Comparison with total glutathione levels revealed that reduced GSH similarly increases in parallel with growth, reaching maximal levels during stationary phase (Fig. 2C). In contrast, oxidized GSSG levels were relatively unaffected in response to growth phase. These data indicate that the increase in GSSP levels observed during the growth cycle does not simply arise in response to a shift in the glutathione redox balance to a more oxidized state.

**Figure 3:**
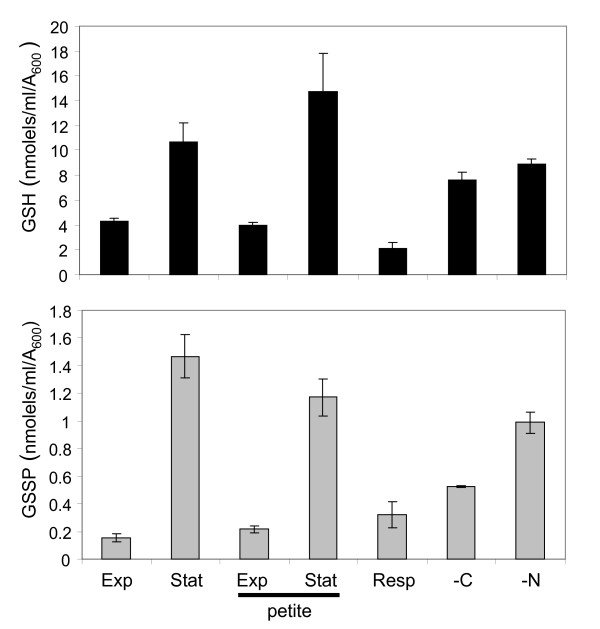
**Increased stationary phase glutathionylation is a starvation response**. Wild-type and petite mutant strains were grown to exponential (Exp) or stationary (Stat) phase in minimal SD media. The effect of respiratory growth (Resp) was determined by growing the wild-type strain to exponential phase on minimal media containing glycerol and ethanol as carbon sources. Carbon (-C) and nitrogen (-N) starvations were induced by growing the wild-type strain in SD media to exponential phase and shifting cells to fresh media lacking a carbon or nitrogen source, respectively. Total (GSH) and protein bound (GSSP) glutathione levels were determined. Values shown are the means of at least three independent determinations and are given in nmol/ml/A_600_.

The intracellular signal for increased glutathionylation may arise due to the shift to respiratory growth conditions which would be expected to generate ROS via the mitochondrial electron transport chain. To test whether respiratory growth conditions affect glutathionylation, GSSP levels were measured in an isogenic petite [*rho*^0^] mutant which is unable to grow by respiration (Fig. 3). A stationary phase increase in glutathionylation was still observed in this mutant indicating that the shift to respiratory growth does not account for increased glutathionylation. To test directly whether respiratory growth elevates glutathionylation, GSSP levels were examined in exponential phase cells grown on a respiratory carbon source (glycerol and ethanol). GSSP levels were moderately elevated by approximately two-fold under respiratory conditions, but were still significantly lower than that observed in stationary phase cells (Fig. 3). Similarly, rapidly shifting cells from a fermentative to a respiratory carbon source did not increase glutathionylation to stationary phase levels (data not shown). Since stationary phase occurs when cells exhaust essential nutrients, we tested whether glutathionylation is increased in response to carbon or nitrogen starvation. Cells were grown to exponential phase and shifted to media lacking glucose or a nitrogen source for four hours. In both cases, GSSP levels were elevated with a more pronounced five-fold induction observed in response to nitrogen starvation (Fig. 3). Similar to the growth phase regulation of GSSP levels, total glutathione levels were also increased in response to nutrient starvation. Thus, the increase in stationary phase glutathionylation appears to predominantly be a response to starvation conditions.

### Glutathionylation is constitutively high in thioredoxin mutants

**Figure 4:**
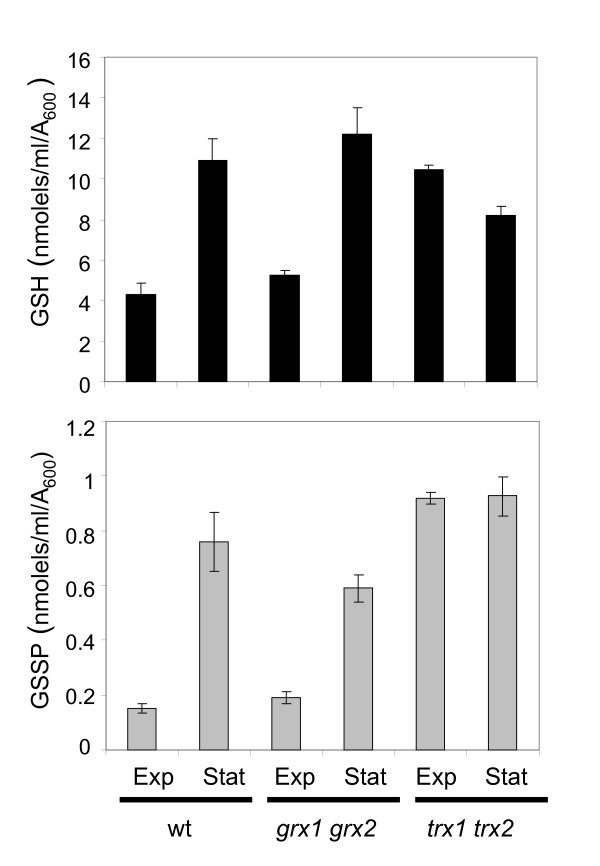
**Thioredoxins are required to maintain low levels of glutathionylation during exponential phase growth**. The wild-type, glutaredoxin (*grx1 grx2*) and thioredoxin (*trx1 trx2*) mutant strains were grown to exponential (Exp) or stationary (Stat) phase in minimal SD media. Total (GSH) and protein bound (GSSP) glutathione levels were determined. Values shown are the means of at least three independent determinations and are given in nmol/ml/A_600_.

The low levels of glutathionylation observed during exponential phase growth may simply arise due to the lack of any signal to elevate GSSP levels, or alternatively, GSSP levels may by kept low by an enzymatic activity. As described in the Introduction, glutaredoxins have been proposed to be the predominant deglutathionylase enzymes in many systems. We therefore examined the growth phase-dependent regulation of glutathionylation in mutants lacking glutaredoxins. GSSP levels in a glutaredoxin mutant (*grx1 grx2*) were comparable to the wild-type control strain, and displayed a similar stationary phase increase (Fig. 4). In contrast, analysis of GSSP levels in a thioredoxin mutant (*trx1 trx2*) revealed that they are constitutively high during both exponential and stationary growth phases (Fig. 4). Similarly, total glutathione levels were elevated in the *trx1 trx2 *mutant consistent with the known constitutive activation of the Yap1 transcription factor which induces the expression of a number of antioxidants including glutathione biosynthetic enzymes in thioredoxin mutants [20]. These data indicate that thioredoxins appear to be required to maintain low levels of GSSP during exponential growth.

**Figure 5:**
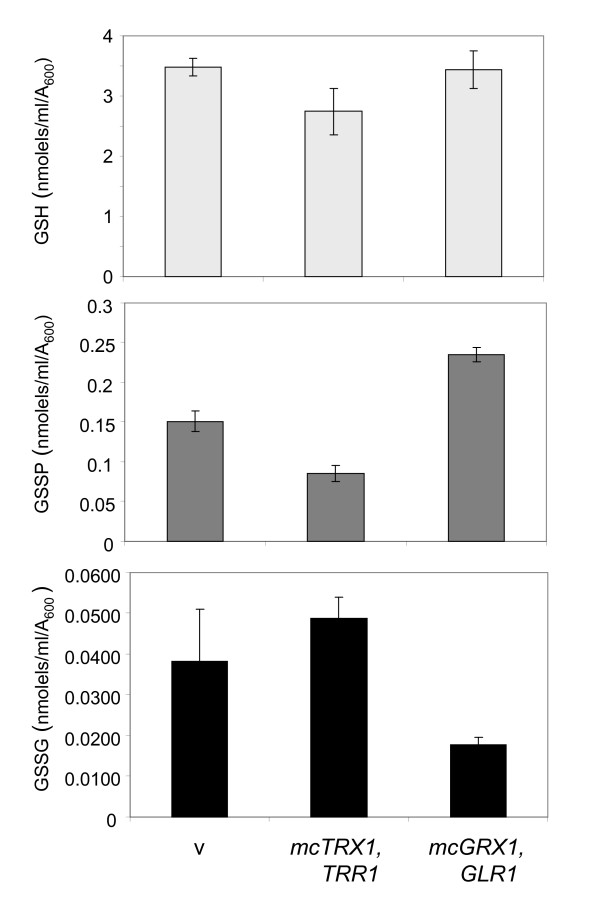
**Overexpression of the thioredoxin system reduces glutathionylation**. The wild-type strain containing pRS426 (v), thioredoxin system (Trx1 and Trr1) or glutaredoxin system (Grx1 and Glr1) was grown to exponential phase in minimal SD media. Reduced (GSH), oxidized (GSSG) and protein bound (GSSP) glutathione levels were determined. Values shown are the means of at least three independent determinations and are given in nmol/ml/A_600_.

In a further effort to define the role of glutaredoxins and thioredoxins in protein glutathionylation, we examined whether their overexpression alters GSSP levels (Fig. 5). Multi-copy vectors were used to overexpress the thioredoxin (*TRX1, TRR1*) or glutaredoxin (*GRX1, GLR1*) systems. We have previously used these vectors to confirm that overexpression of *GRX1 *and *TRX1 *confers resistance to hydrogen peroxide [17]. Overexpression of the thioredoxin system did not significantly affect the levels of oxidized (GSSG) or reduced (GSH) glutathione (Fig. 5). In agreement with the idea that thioredoxins maintain low exponential phase GSSP levels, overexpression of the thioredoxin system was found to decreases GSSP levels by approximately 50%. In contrast, overexpression of the glutaredoxin system resulted in an approximate 66% increases in GSSP levels, confirming that glutaredoxins do not act as general deglutathionylases *in vivo*. GSH levels were unaffected, whereas, GSSG levels were significantly lowered by the glutaredoxin system presumably due to the activity of Glr1. These data further confirm that there does not appear to be any strong correlation between cellular GSSP and GSSG levels.

### Thioredoxins, but not glutaredoxins, are active as deglutathionylases

**Figure 6:**
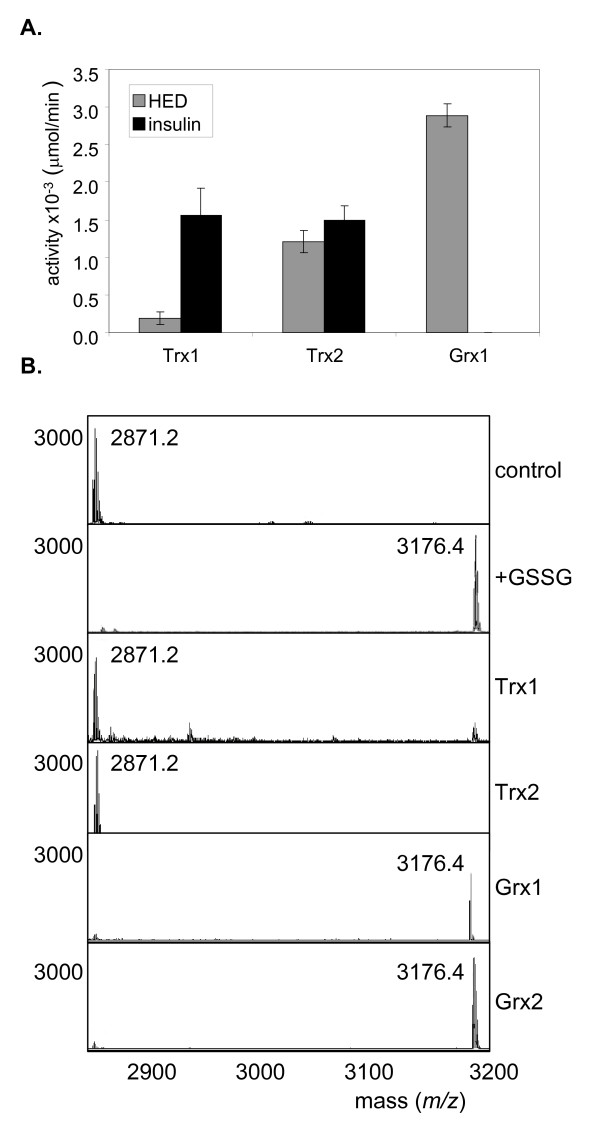
**Thioredoxins reduce glutathionylated proteins *in vitro***. **(A) **Thioredoxin and glutaredoxin activities for purified proteins were determined using standard enzyme assays. Oxidoreductase activity was measured by the ability to reduce the mixed disulphide formed between GSH and HED. The reaction rate for Grx1 was followed by the oxidation of NADPH by glutathione reductase. Thioredoxins were tested in this assay by substituting Grx1 and Glr1, with Trx1 or Trx2 and Trr1, respectively. Protein disulphide reductase activity was measured using insulin as a substrate. The reduction of disulphide bonds in insulin was followed by the oxidation of NADPH by thioredoxin reductase (Trx1 and Trx2) or glutathione reductase (Grx1). Reaction rates are given as μmoles of NADPH oxidized per minute (×10^-3^) and represent the means of at least three independent determinations. **(B) **MALDI-TOF analysis of glutathionylated creatine kinase. The ion at 2871.2 m/z corresponds to the peptide containing the active site cysteine residue (control). Incubation with a 10-fold molar excess of GSSG (+GSSG) resulted in a new peak with a mass shift of 305 Da (3176.4 m/z) consistent with the addition of a glutathione molecule. Thioredoxins (Trx1 or Trx2) can reduce glutathionylated creatine kinase and the modified peptide was shifted to the unmodified form. In contrast, glutaredoxins (Grx1 or Grx2) did not display deglutathionylase activity and the glutathionylated peptide was still detected (3176.4 m/z)

Thioredoxin and glutaredoxin system components were purified to confirm their roles in deglutathionylation. Well-established glutaredoxin and thioredoxin-dependent assays were first used to confirm that the purified proteins were active. The oxidoreductase activity of glutaredoxins has been extensively characterized by their ability to reduce the mixed disulphide formed between GSH and HED [21]. In this reaction, glutaredoxins reduce the GSH-HED mixed disulphide and the reaction rate is followed by the oxidation of NADPH by glutathione reductase. Purified Grx1 was active in this assay confirming GSH-dependent oxidoreductase activity (Fig. 6A). Thioredoxins were also tested in this assay by substituting Grx1 and Glr1, with Trx1 or Trx2 and Trr1, respectively. The yeast thioredoxins were active in this assay, albeit with reduced activity compared to Grx1. Interestingly, Trx2 was more active than Trx1 suggesting a difference in the mixed disulphide oxidoreductase activities of the yeast thioredoxin isoenzymes. The protein disulphide reductase activity of thioredoxins is often measured using insulin as a substrate [22]. In this reaction, thioredoxins reduce disulphide bonds in insulin and the reaction is followed by the oxidation of NADPH by thioredoxin reductase. Trx1 and Trx2 were both active in this assay and displayed similar reaction rates (Fig. 6A). In contrast, no reactivity was found with Grx1 (in a reaction containing Glr1 and GSH) indicating that Grx1 is unable to reduce protein disulphides.

We directly examined deglutathionylase activity using two model glutathionylated substrates. Creatine kinase is a well characterized enzyme which can undergo glutathionylation at its active site cysteine residue [23]. We incubated rabbit creatine kinase with a ten-fold molar excess of oxidized GSSG and examined glutathionylation by tryptic digestion and MALDI-TOF-MS analysis (Fig. 6B). The tryptic peptide encompassing the active site (2871 m/z) was found to show a mass increase of 305 Da (3176.4 m/z) consistent with a single modification by glutathionylation. Incubation with the glutaredoxin system (Grx1 or Grx2, GSH, Glr1) did not affect the glutathionylated peptide. In contrast, both Trx1 and Trx2 were able to reduce the glutathionylated peptide to the non-modified form. To confirm that the reactivity of the thioredoxin system is not confined to creatine kinase, deglutathionylase activity was also tested with glyceraldehyde 3-phosphate dehydrogenase (GAPDH) which has frequently been identified as a target of glutathionylation in various cellular systems [24]. Glutathionylation of the active site Cys residue of GAPDH was similarly detected by tryptic digestion and MALDI-TOF-MS analysis (data not shown). The thioredoxin system (Trx1 or Trx2) was able to reduce glutathionylated GAPDH, whereas, no glutathionylase activity was detected with the glutaredoxin system. These data confirm that yeast cytoplasmic thioredoxins, but not glutaredoxins, are active in deglutathionylation.

### Thioredoxin mutants are delayed in the transition from stationary phase to exponential phase growth

**Figure 7:**
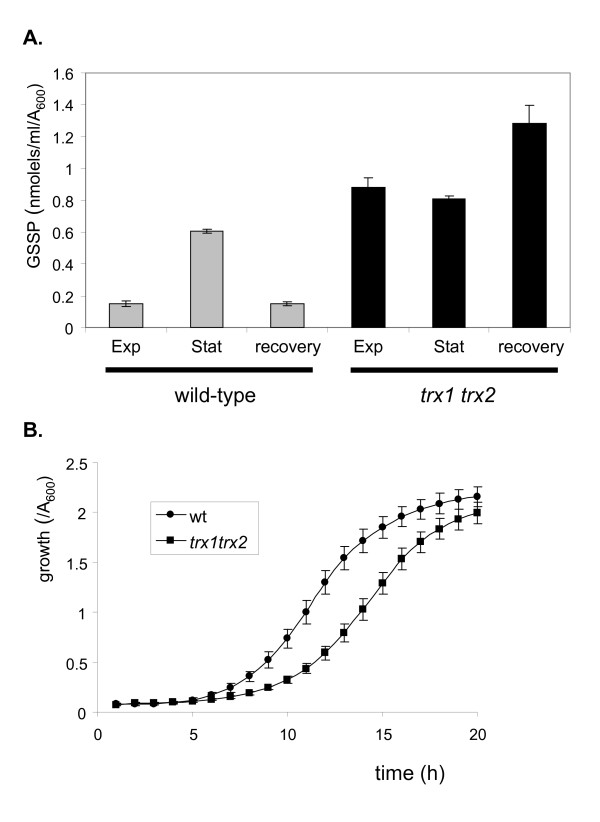
**Thioredoxins reduce glutathionylated proteins as cells resume exponential phase from stationary phase growth**. **(A) **Thioredoxins are required to reduce glutathionylation during the transition from stationary phase to exponential phase growth. Stationary phase cells of the wild-type and thioredoxin mutant strains were inoculated into fresh SD media and protein bound (GSSP) glutathione levels determined after two hours growth. Values shown are the means of at least three independent determinations and are given in nmol/ml/A_600_. **(B) **Thioredoxin mutants are delayed in the exit from stationary phase. Stationary phase cells of the wild-type and thioredoxin mutant strains were inoculated into fresh SD media and growth monitored by following A_600_.

Given that protein glutathionylation was found to be maximal during stationary phase, we reasoned that deglutathionylation must occur when cells exit stationary phase and re-enter exponential phase growth. We therefore examined glutathionylation in stationary phase cells, following inoculation into fresh media, containing all the necessary nutrients to resume growth. The high levels of stationary phase glutathionylation were reduced within two hours in a wild-type strain (Fig. 7A). In contrast, glutathionylation was constitutively high in a thioredoxin mutant confirming that thioredoxins are required for deglutathionylation. To test whether the lack of deglutathionylation activity affects the resumption of growth in stationary phase cells, we compared the growth cycle of wild-type and thioredoxin mutants. Cells were inoculated from stationary phase cultures into fresh media, at identical starting cell densities (A_600 _= 0.2). This analysis demonstrated that there is a longer lag phase in thioredoxin mutants which take approximately 11 hours to enter exponential growth, compared with eight hours in the wild-type control strain (Fig. 7B). Once exponential phase resumed, the thioredoxin mutant was relatively unaffected and displayed a similar doubling time to the wild-type strain.

## Discussion

Yeast, like most eukaryotes, contains a complete cytoplasmic thioredoxin system, which functions in protection against oxidative stress. Trx1 and Trx2 are active as antioxidants and play key roles in protection against oxidative stress induced by various ROS [17]. Grx1 and Grx2 are active as GSH-dependent oxidoreductases, but appear to have distinct cellular functions [18,19]. Five monothiol glutaredoxins have also now been identified in yeast, differing from classical glutaredoxins in that they contain a single cysteine residue at their putative active sites. They are found in different subcellular compartments including nuclear (Grx3-4), the mitochondrial matrix (Grx5) and the early secretory pathway (Grx6-7) [25-27]. Grx5 has been proposed to play a role in protein glutathionylation and is required for deglutathionylation of cytosolic GAPDH [28]. However, *grx5 *mutants are defective in the assembly of Fe/S enzymes and the mitochondrial localization of Grx5 means that any effect on deglutathionylation is most likely indirect caused by the alteration in cellular iron levels [29].

Glutaredoxins have been proposed to be the predominant deglutathionylase enzymes in many eukaryotic systems. However, analysis of GSSP levels in yeast glutaredoxin mutants (*grx1 grx2*) revealed that they are comparable to the wild-type control strain. Additionally, we found that glutaredoxins were unable to reduce model glutathionylated proteins *in vitro*. It is surprising that the yeast glutaredoxins do not appear to influence protein glutathionylation since this is one of the main activities described for these enzymes in many mammalian and plant systems. For example, *in vitro *comparisons using glutathionylated substrates revealed that glutaredoxin, thioredoxin, protein disulphide isomerase, glutathione, and cysteine all display deglutathionylation activity, but glutaredoxin was found to be the most efficient deglutathionylase enzyme [15,30]. In addition, a correlation between protein-SSG reduction and glutaredoxin activity has been demonstrated in mammalian cells [13] and the reversible S-glutathiolation of HIV-1 protease can be catalysed by a glutaredoxin *in vitro *[5]. Grx1-knockout mice have been constructed and are deficient in deglutathionylase activity, but were surprisingly unaffected in sensitivity to oxidative insults [31]. Perhaps the most compelling evidence for a role of human Grx1 in deglutathionylation has come from studies where altered levels of Grx1 have been shown to regulate glutathionylation of several specific target proteins including Ras, inhibitory kappa B kinase, actin and caspase 3 [32-35]. We cannot at this stage rule out that the yeast glutaredoxins are required for deglutathionylation of specific target proteins which would not be detectable in our analysis of global modification levels. Interestingly, mammalian mitochondrial glutaredoxin 2 has been implicated in protein glutathionylation, catalysing the formation of protein mixed disulphides with glutathione [14]. We similarly observed that overexpression of yeast Grx1 elevates global protein glutathionylation levels. This did not arise due to a shift to a more oxidizing environment since oxidized GSSG concentrations were lowered in parallel with the increase in glutathionylation. Further work will be required to determine whether yeast glutaredoxins catalyse glutathionylation of specific target proteins.

Unlike in glutaredoxin mutants, high levels of glutathionylation were detected in thioredoxin mutants (*trx1 trx2*). Purified thioredoxins were also found to catalyse deglutathionylation of model substrate proteins. The requirement for yeast thioredoxins to maintain protein glutathionylation levels is in contrast to thioredoxins from other eukaryotic species which are generally thought to be inefficient in deglutathionylation [4,15,36]. The low levels of glutathionylation detected during exponential phase growth could be further reduced by overexpression of the thioredoxin system indicating that this protein modification is constitutively present on at least some target proteins. We found that protein glutathionylation levels peak in wild-type cells as they exit exponential phase and enter stationary phase growth. In contrast, glutathionylation was constitutively high during all growth phases in thioredoxin mutants. Our data indicate that thioredoxin activity appears to be required to reduce the high levels of stationary phase glutathionylation as cells exit this growth phase and resume exponential phase growth. This requirement for thioredoxins correlates with the increased expression of *TRX1 *and *TRX2 *which is observed in stationary phase cells [17].

Glutathionylation has been proposed to serve a protective function which prevents the irreversible oxidation of cysteine residues during oxidative stress conditions. However, our data indicate that the stationary phase increase in glutathionylation is unlikely to arise due to a simple increase in ROS generated by respiratory growth since glutathionylation was only modestly increased in cells grown on a respiratory carbon source and was unaffected in respiratory deficient cells. Additionally, little or no increase in oxidized GSSG was detected indicating that glutathionylation does not correlate with a shift in the glutathione redox couple to a more oxidized state. Glutathionylation was significantly increased in response to starvation for carbon or nitrogen which may indicate that this protein modification is a general response to starvation conditions. The increase in glutathionylation was coincident with maximal levels of cellular glutathione which are detected as cells exit stationary phase [37]. This may suggest that protein-bound glutathione serves as a store which can be rapidly mobilized when cells resume active growth. This idea is supported by the observation that there appears to be a correlation between the levels of reduced GSH and GSSP, which are increased in parallel in thioredoxin mutants and in response to starvation conditions. GSSP levels may therefore reflect an in increase in the reactants of glutathionylation, rather than a physiologically controlled process. However, the finding that overexpression of the glutaredoxin system elevates GSSP levels without altering GSH levels, argues against this idea since increased glutathionylation is observed in the absence of increased GSH levels. An alternative possibility is that glutathionylation may serve a regulatory role which alters the activity and/or structure of cysteine-containing proteins. For example, glutathionylation inhibits the activity of a number of glycolytic enzymes which are not required in stationary phase cells in the absence of active glucose-based growth [3]. Little is known regarding the metabolic changes that occur in lag phase cells as they resume vegetative growth following exit from stationary phase. Our data indicate that thioredoxin mutants are delayed in lag phase, but they eventually resume exponential phase growth following the restoration of nutrient rich conditions. This may mean that essential enzymes are inhibited by glutathionylation in thioredoxin mutants, and the delayed resumption of growth is due to the requirement to synthesize new active enzymes.

## Conclusions

This study has shown that thioredoxins, and not glutaredoxins, are required to maintain protein glutathionylation levels during the yeast growth cycle. This is in contrast to other eukaryotic systems where glutaredoxins appear to be the predominant deglutathionylase enzymes. Our data add to the growing evidence indicating a functional overlap between the GSH/glutaredoxin and thioredoxin systems. Redox-active proteins which are modified by the addition of glutathione can be reversibly regulated by the thioredoxin system, providing a mechanism to coordinate regulation by the two major cellular redox regulatory systems.

## Methods

### Yeast Strains, plasmids and growth conditions

The *Saccharomyces cerevisiae *strains used in this study were isogenic derivatives of W303 (*MATa ura3-52 leu2-3 leu2-112 trp1-1 ade2-1 his3-11 can1-100*). Strains deleted for thioredoxins (*trx1::TRP1 trx2::URA3*), glutaredoxins (*grx1::LEU2 grx2::HIS3*) and an isogenic petite strain have been described previously [17,18,38]. For overexpression studies, multi-copy plasmids containing *GRX1, GLR1, TRX1 *and *TRR1 *were constructed in pRS-based plasmid [39].

Strains were grown in rich YEPD medium (2% w/v glucose, 2% w/v bactopeptone, 1% w/v yeast extract) or minimal SD medium (0.17% w/v yeast nitrogen base without amino acids, 5% w/v ammonium sulphate, 2% w/v glucose) supplemented with appropriate amino acids and bases [40] at 30°C and 180 rpm. For growth on non-fermentable carbon sources, SGE contained 3% (v/v) glycerol and 1% (v/v) ethanol. Nitrogen (N) starvation medium, contained 2% (w/v) glucose, 0.17% (w/v) yeast nitrogen base without amino acids and limited amounts of auxotrophic requirements (1 mg/liter for tryptophan and 5 mg/liter for all other cases). For carbon (C) starvation conditions, SD medium was used without glucose. Media were solidified by the addition of 2% (w/v) agar.

### Determination of glutathione levels

Glutathione levels were determined as described previously [41]. Briefly, cells were harvested by centrifugation, washed with phosphate-buffered saline (pH 7.4) to remove any traces of growth medium, and resuspended in ice-cold 8 mM HCl, 1.3% (w/v) 5-sulfosalicyclic acid. Cells were broken with glass beads using a Minibead beater (Biospec Scientific, Bartlesville, OK) for 30 s at 4°C, before incubating on ice for 15 min. to precipitate proteins. Cell debris and proteins were pelleted in a microcentrifuge for 15 min (13,000 rpm 4°C) and the supernatant used for the determination of free glutathione. For quantification of oxidized glutathione (GSSG), samples were pretreated with 5% (v/v) 2-vinylpyridine for 1 h at room temperature before analysis. To release protein-bound glutathione, the pellets from the sulfosalicyclic acid extraction were resuspended in 1% sodium borohydride. To aid the release of GSH, extracts were again shaken on a Minibead beater (20 s., 4°C) before microfuging at 10,000 rpm for 1 h at room temperature. The resulting supernatant was neutralized with 100 mM potassium phosphate buffer pH 7.4 and used to determine protein-bound GSH (GSSP). GSH levels are expressed as nmoles of GSH per 1 A_600 _of cells.

### Protein purification and enzyme assays

Plasmids expressing six-histidine-residue tagged versions of Grx1 (pBAD-YGRX1), Trx1 (pBAD-YTRX1) and Trr1 (pBAD-YTRR1) were a kind gift from Barry Rosen [42]. Trx2 was amplified by PCR and cloned into the pBAD expression vector (Invitrogen). Histidine-tagged proteins were purified by Ni^2+^-NTA chromatography and protein purity checked on SDS-PAGE gels.

GSH-dependent disulphide oxidoreductase activity was measured by the reduction of the mixed disulfide formed between β-hydroxyethylene disulphide (HED) and GSH [21]. To assay Grx1, the reaction mix contained NADPH (0.4 mM), GSH (1.0 mM), glutathione reductase (6 μg/ml) and HED (1.4 mM) in 0.1 M Tris HCl, pH 7.4. A mixed disulphide between HED and GSH is formed within 2 min, and the reaction was started by the addition of 17 μM Grx1. The reaction was followed by the decrease in *A*_340 _due to the oxidation of NADPH. To assay thioredoxins, Grx1 and Glr1 were substituted with Trx1 or Trx2 (1.5 μM) and Trr1 (0.5 μM), respectively. Protein disulphide reduction activity was measured using insulin as a substrate. Reaction mixtures contained insulin (0.6 mg/ml), NADPH (0.6 mM), EDTA (1 mM), Trx1 or Trx2 (1.5 μM) and Trr1 (0.5 μM) in 25 mM Tris HCl, pH 8.0. To assay glutaredoxin, Trx1 or Trx2 and Trr1, were substituted with Grx1 and Glr1 respectively. The reaction was followed by the decrease in *A*_340 _due to the oxidation of NADPH.

### Mass spectrometry analysis

Creatine kinase and GAPDH were glutathionylated as previously described [43]. Peptides were analysed by matrix-assisted laser desorption ionization/time-of-flight mass spectrometry (MALDI-TOF-MS) on a Bruker Ultraflex II mass spectrometer. The instrument was calibrated externally with peptide standard II from Bruker, resulting in a mass accuracy of 100 ppm in the range up to 5000 Da.

## Authors' contributions

DG performed mass spectrometry, growth analysis and glutathione assays. JV carried out the purification of the thioredoxin and glutaredoxin system proteins and performed insulin and HED assays. DS and GP performed the glutathione assays. ID and CG conceived of the study, and participated in its design and coordination. CG drafted the manuscript and all authors read and approved the final manuscript.
